# Addressing “what matters most” to reduce mental health stigma in primary healthcare settings: a qualitative study in Lebanon

**DOI:** 10.1186/s12875-024-02680-2

**Published:** 2024-12-19

**Authors:** Racha Abi Hana, Eva Heim, Pim Cuijpers, Marit Sijbrandij, Rabih El Chammay, Brandon A. Kohrt

**Affiliations:** 1 National Mental Health Programme, Ministry of Public Health, Beirut, Lebanon; 2 Department of Clinical, Neuro- and Developmental Psychology, Vrije Universiteit, Amsterdam, The Netherlands; 3 Institute of Psychology, University of Lausanne, Lausanne, Switzerland; 4 Department of Psychiatry, Saint Joseph University, Beirut, Lebanon; 5 Center for Global Mental Health Equity, Department of Psychiatry and Behavioral Health, George Washington University, Washington, DC USA

**Keywords:** Stigma, Primary care, Developing countries, Mental health, Education, Training

## Abstract

**Background:**

“What matters most” (WMM) is a theoretical framework based on medical anthropology and draws on cultural concepts of values and morals. It has been employed to identify cross-cultural aspects of mental health stigma. This approach assists practitioners, advocates, and researchers in assessing stigma-related factors that are relevant to the experiences of individuals in diverse cultural contexts. To implement effective anti-stigma programmes it is vital to identify and prioritize WMM for primary healthcare providers and people with lived experience of mental health conditions (PWLE). Our current objective was to explore WMM to primary healthcare providers, PWLE, primary care managers, and policymakers in Lebanon to inform mental health stigma reduction initiatives.

**Methods:**

We conducted a total of 45 qualitative interviews with primary healthcare providers, PWLE, primary care managers, and policymakers. The WMM framework was applied to analyse data from primary healthcare centres in Lebanon to identify themes related to stigma against PWLE. The analysis identified common themes related to WMM. The analysis aimed to identify (a) WMM values for participants, (b) factors that threaten these WMM values and their relationship to stigma, and (c) potential interventions that could leverage WMM principles to reduce stigma.

**Results:**

WMM for primary healthcare providers encompassed competency, time management, willingness, and self-care. WMM for PWLE focused on equality, support, compassion, and confidentiality. Policymakers emphasised resource sustainability as a top priority. Myths about mental health illnesses perpetuated threats to WMM, and organisational barriers also threatened WMM for primary healthcare providers and PWLE, thus creating major roadblocks to achieving stigma reduction.

**Conclusion:**

This study identified key domains to understand the factors for WMM in reducing mental health stigma in Lebanon and explored factors that shape the values and priorities of both PWLE and primary healthcare providers. The study suggests assessing the effectiveness of anti-stigma interventions that actively engage PWLE in their design and implementation, while exploring the broader applicability of the WMM framework across different cultural and healthcare settings.

**Supplementary Information:**

The online version contains supplementary material available at 10.1186/s12875-024-02680-2.

## Background

Stigma refers to a mark of shame that often prompts negative perceptions and attitudes towards those who bear it; this includes people with lived experience of mental health conditions (PWLE) [1]. Stigma can lead to discriminatory behaviour, which can have even more severe consequences than the mental health conditions [2]. Mental health stigma is linked to poor healthcare delivery, including insufficient screening, diagnosis, and treatment that resulted in early mortality, particularly in low- and middle-income countries (LMICs) [3].

The “what matters most” (WMM) theoretical framework derives from medical anthropology research related to local values and morals. Within this framework, behaviours that are stigmatised in a given context are those that threaten values and morals related to what matters most to be a member of that society [4]. It has been employed to identify cross-cultural aspects of mental health stigma [5]. It entails recognising culture-specific features of mental health stigma by emphasising the characteristics that are most important within a given cultural context [5]. This allows for a more nuanced understanding of how stigma affects individuals in various cultural situations [5].

Building on this understanding, the WMM framework has been adapted to analyse cultural differences in the stigmatisation of mental health conditions across various societies [6]. The WMM theoretical framework suggests that stigmatization arises when behaviours or conditions are perceived as threats to the core values and priorities of a social group. In other words, threats to what a society values most lead to discrimination and stigma [5]. For instance, in capitalist societies, economic productivity is often a central value, and thus, the perceived lack of economic productivity among individuals with mental health conditions is viewed as a threat, resulting in stigmatization [7]. In healthcare settings, the competence of providers and their ability to alleviate suffering are fundamental values, meaning that patients who are perceived as untreatable are seen as threatening these values, leading to stigma directed at people living with mental illness [8]. Across all cultures, the preservation of life and survival is universally considered fundamental, which means that violence is perceived as a significant threat. As a result, perceptions of violence associated with mental illness further contribute to stigmatization. Ultimately, the WMM framework emphasizes the importance of identifying the values that matter most to a given group, understanding how perceived threats to these values lead to stigma, and developing interventions that address and challenge the beliefs underlying these threats [8]. Adaptation of stigma interventions direct research efforts towards areas that may have been previously overlooked and helps in implementing effective anti-stigma approaches by prioritising culturally relevant issues [5].

As Yang et al. [5] highlight, rather than solely asking “What do you value?“, the concept of WMM is also observable through everyday actions—essentially, “What do you do?“. It has been suggested that culture influences stigma by threatening an individual’s ability to engage in activities that are central to defining WMM or ‘personhood’ within a specific cultural context [5]. According to Knaak et al. [9], a qualitative synthesis of successful anti-stigma interventions emphasized the value of various forms of social contact as a crucial component in questioning and changing negative beliefs in society. According to the data compiled by the Lancet Commission on ending stigma and discrimination in mental health, the voices of those who have really experienced mental health issues were the main agents of change for reducing stigma [2]. A recent study by Kohrt et al. [8] offered important insights into the successful reduction of stigma in healthcare settings, demonstrating that stigma reduction interventions were most successful when social interaction targets WMM to the stigmatizing group. Sociocultural context impacts how individuals within a cultural group experience stigma [10]. For example, in China, stigma related to mental illness can impact individuals’ social standing and honour, while in the United States, stigma associated with first-onset schizophrenia often affects individuals’ emotional well-being and social roles [10]. Stigma varies across cultures, and according to Schomerus [6], these differences can be understood by identifying WMM within a cultural context and contrasting it with WMM in another culture. Kirmayer and Pedersen [11], highlighted that interventions often disregard non-Western perspectives which can limit their effectiveness. Mascayano’s [12] study underscored that only a minority of stigma reduction interventions address cultural values and practices, indicating a gap in culturally tailored approaches.

In light of these insights, Lebanon, a middle-income nation, is confronted with a critical mental health treatment gap that exceeds ninety percent [13]. The healthcare system has been overwhelmed by the combination of political instability and the influx of people arising from the Syrian crisis [14]. The national mental health strategy launched by the National Mental Health Programme (NMHP) at the Ministry of Public Health (MOPH) in Lebanon highlights stigma as a major impediment to mental health treatment utilization [15]. To address obstacles like financial limitations and structural problems, the NMHP worked with partners to incorporate mental health services into a subset of primary healthcare centres (PHCs). A previous qualitative study done in primary healthcare centres in Lebanon revealed that stigma was still a key concern that affected PWLE [16]. Similarly, Noubani et al. [17] used a community-based system dynamics approach to examine factors affecting mental health and health-seeking behaviours in Lebanon, finding that participants consistently reported substantial stigma faced by PWLE. This stigma not only worsens public perception of mental health conditions but also exacerbates challenges within Lebanon’s already fragile health system, underscoring the need for comprehensive multi-sectoral efforts to reduce stigma and improve integrated, person-centred care [18].

Our current objective was to explore the WMM framework among PWLE, healthcare providers (HCPs), policymakers, and management in PHCs in Lebanon to inform anti-stigma interventions that are culturally grounded. Although the previous study [16] identified the presence of stigma in Lebanon, our current research aims to build on this initial understanding by offering detailed guidance on culturally adapted anti-stigma interventions. We focus on identifying and addressing WMM based on different respondent groups. This objective was extremely significant, as it goes beyond just recognising stigma and takes direct steps to address it with appropriate measures.

## Method

### Study design

This study was a part of a larger multinational study called INDIGO-PRIMARY, which focused on examining mental health stigma in primary care across seven countries [19]. In the INDIGO project, the “PRogramme for Interventions addressing Mental healthcare knowledge, Attitudes, and behaviour in primaRY care” (PRIMARY) initiative was designed to create, implement, and assess interventions aimed at enhancing the knowledge, attitudes, and behaviours of primary care providers in their interactions with PWLE in primary care settings [19].

The methods employed included the analysis of previous data sources to identify the WMM framework to reduce stigma against PWLE at primary healthcare centres in Lebanon. While our preliminary analysis focused on the general perspectives of different respondent groups [16], this study delves deeper using the WMM framework to analyse and understand the identified themes comprehensively. We retrospectively applied the WMM theoretical framework to this previously collected data to identify (a) WMM to healthcare providers, PWLE, PHC management, and policymakers; (b) what threatens WMM values and this relationship to stigma; and (c) what interventions could be done that build upon WMM in this setting.

### Participants

The pre-existing qualitative dataset includes semi-structured interviews with policymakers, PHC management, HCP, and PWLE. The NMHP team and MOPH PHC section worked together to choose four PHC. Qualitative interviews (*n* = 45) were conducted between August 2018 and December 2019 with programme managers and policymakers (*n* = 3), PWLE (*n* = 14), nurses (*n* = 6), general practitioners (GPs) (*n* = 5), mental healthcare professionals (*n* = 6), frontline practitioners (*n* = 4), PHC management (*n* = 4), and other staff (*n* = 3). The demographics and additional details of the participants are detailed in the previous study [16].

### Sampling strategy

Selection of four PHCs was done in collaboration with the PHC department at MOPH and the NMHP team. The selection criteria for these centres included having staff trained in mental health care through the mental health Gap Action Programme (mhGAP), a high patient load to ensure a sufficient number of PWLE could be interviewed, and the availability of mental health professionals for interviews. Additionally, the centres were located in Beirut and Mount Lebanon to facilitate convenience and access within these urban areas.

### Recruitment

The NMHP team coordinated with focal persons in each centre, such as the centre directors or management coordinators, who were responsible for the initial contact with key informants and PWLE. Participants were chosen based on availability and their identification with one of the key groups involved in the study. No additional specific sampling methods were applied beyond these criteria.

### Data collection tool

The interview guides were created earlier for the INDIGO-PRIMARY project based on five distinct topic guidelines [19]. The questions were adapted to meet the Lebanese context, taking cultural and contextual subtleties into consideration [16]. The topic guides included a list of topics, general questions, and probes for investigation. All questionnaires were translated into Arabic. The questionnaires were internally tested by NMHP staff and adjusted as needed. Study participation began upon giving written informed consent. Prior to the interview, the interviewer ensured that the participant had sufficient opportunity to ask any clarifying questions. Phone interviews were primarily conducted for PWLE who requested them, and in these instances, verbal consent was obtained.

### Ethics

The ethics protocol to conduct initial interviews was approved from Saint Joseph’s University Beirut (CEHDF 1193). The complete specifications for the Consolidated Criteria for Reporting Qualitative Studies (COREQ) can be found in the supplemental file 1. It includes the 32-item checklist for consolidated criteria for reporting qualitative studies (COREQ) [20].

### Data analysis

The transcribed data was meticulously examined for recurring themes and issues. These were categorized according to their relevance to WMM from the perspectives of healthcare providers, PWLE, centres management and policymakers. Repetitive issues were then grouped under broader domains such as confidentiality, equality, stigma, etc. The thematic analysis aimed to highlight significant areas impacting the delivery of mental health services within the PHC setting. This approach was chosen to give a complete understanding of the underlying causes of mental health stigma in Lebanon, as well as to inform the creation of tailored interventions to reduce stigma in primary healthcare settings. With the WMM framework, the analysis focused on identifying the most significant themes that emerged from the data—those that participants identified as central to their decision-making, values, and behaviours. Moreover, the analysis sought to highlight key concerns and priorities across different key groups, ensuring that the experiences and perspectives of healthcare providers, PWLE, centres management, and policymakers were all considered. In addition, the established framework was applied to identify potential threats to the values of WMM. This involved a detailed assessment of barriers and challenges mentioned during the interviews. The initial analysis, conducted by the first author, focused on identifying and organizing key themes to WMM, threats and barriers to the values and priorities of WMM. This analysis was reviewed by the senior author to ensure accuracy, consistency, and reliability.

## Results

**Fig. 1:**
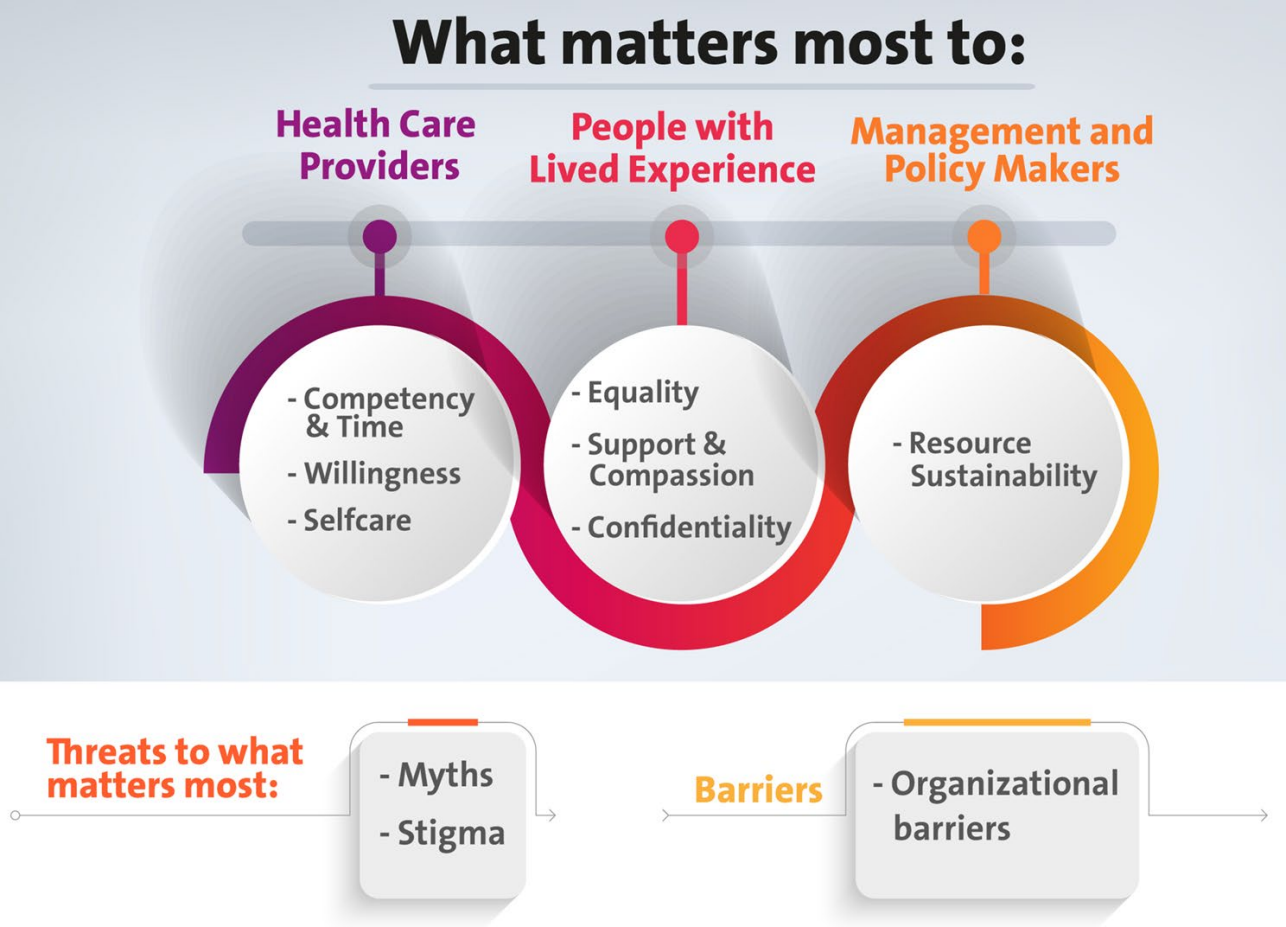
Conceptual framework for what matters most in Lebanon. (Adapted from Gurung et al. [21])

In the results section, we present a comprehensive list of WMM derived from the qualitative analysis. These themes, categorized by domain, highlight key concerns and priorities across different key groups. The insights are organized into several major themes: Equality Concerns, Support and Compassion, Confidentiality, Competency and Time Management, Willingness, Self-Care and Burnout, Resource Sustainability, Perceptions of Stigma, Perceptions of Myths in Mental Health, and Organizational Barriers. The implications for WMM in stigma reduction were addressed in the Table 1. The domains of WMM derived from qualitative data analysis are synthesized and visualized in a conceptual framework (Fig. 1). This framework serves as a tool to comprehend WMM to HCPs, PWLE, and policymakers. Furthermore, it highlights the areas and domains where additional interventions can be strategized to effectively reduce mental health-related stigma in Lebanon.

**Table 1:** Mapping stakeholder perspectives on what matters most in relation to mental health services in primary care

Domain	People with lived experience of mental health conditions (PWLE)	Healthcare providers (HCPs)	Other key stakeholders	Implications for WMM stigma reduction
Equality in health services	Emphasized the importance of being treated by HCPs equally to people with physical health conditions.	Highlighted the importance of maintaining professionalism and treating all PWLE equally regardless of their condition.	Policymakers reinforced the importance of integrating mental health into primary care, aiming to provide equal treatment for mental health disorders and physical disorders.	During training, the shared goal to be highlighted: Equality is important to PWLE experiences, to demonstrate professionalism of providers, and to care systems by policymakers.
Support/ compassion	Underscored that their experiences were improved when HCPs adopted compassionate and responsive approaches, regardless of their mental health conditions.	Expanding their roles to establish a supportive environment for PWLE, noting that staff attitudes and support may vary.	The attitudes of healthcare managers were mixed, and managers were reluctant to support PWLE in distress.	In training sessions, emphasize the common goal by acknowledging compassionate and responsive approaches, incorporating role-playing exercises to hone compassion-building skills and focus on de-escalation techniques.
Confidentiality	Assuring that confidentiality regarding their condition will be maintained by HCPs.	Building trust and assuring confidentiality helps PWLE to disclose about their condition and treatment needs.	While proactive measures are implemented in certain PHCs to safeguard the confidentiality of SU, there is a need for improvement in the structural facilities of other centres.	This could be addressed by structural changes in providing private physical spaces and confidential record keeping in addition to strict policies.
Competency and time	Emphasized that while HCPs were perceived as competent, there was a need for an increased allocation of time to address their concerns and needs effectively.	Having protected time devoted to mental health care. Having adequate training and continuous supervision provided by MOPH to ensure competency.	Policymakers underscored the need for competent HCPs, for more time, and training to be offered for them. Policymakers raised concerns about staff turnover in mhGAP-trained personnel and discontinuity of support and supervision.	Setting up systems that allow for more time for providers to get training, supervision, and deliver MH services which require more allocation of funding.
Willingness		Hesitancy to offer mental health treatment due to concerns about expanding their main responsibility. Preferred to refer mental health issues to specialized professionals and institutions.	Limited management willingness to adjust job descriptions for mental healthcare indicated reluctance to make structural changes and integrate mental healthcare within the PHC.	Collaborative engagement between specialists and primary care workers is crucial, along with policy changes to reinforce the delivery of mental healthcare by HCPs.
Burnout/ Self-care		Burnout was a common occurrence due to long work hours, dealing with PWLE and other stresses like high workloads and poor support.	Some managers challenged the burnout claims validity, believing that working hours are sufficient while acknowledging external and personal stressors. Other managers advocated for increased staff numbers to alleviate the burden on team dynamics.	The need for a comprehensive approach through self-care and support to manage employee burnout, considering variables like staffing, communication, and patient education.
Resource sustainability	Financial barriers to accessing care (e.g., transportation costs, medications cost when out of stock, etc.).	HCPs should be compensated for providing mental health care. Training attendance days should be compensated.	Policymakers emphasized the need for sustainable funding by addressing human resources sustainability, the integration of MH into PHCs.	Ensuring access to care through PHCs as a solution, adequate funding is needed for staffing, integrating mental health, training, medication sustainable provision, the establishment of community mental health services, referral systems, and enhancing overall access to care.
Stigma	Reduced discrimination from HCPs, frontliners and centre management.	Acknowledged that PWLE experienced stigma and discrimination.	PHC Managers displaying stigma towards PWLE contributed to their resistance in integrating mental health services.	Stigma reduction interventions for HCPs must include addressing stigmatizing beliefs and the negative societal attitudes prevalent in the community significantly impacting PWLE’s willingness to seek mental health care.
Misconceptions	Misunderstandings about seeking mental healthcare and using medication for mental health disorders. Certain behaviours were inherent to a person’s personality rather than symptoms of a mental disorder.	Cultural and religious beliefs as per HCPs often contributed to myths and stigma surrounding mental health, connecting them to faith and discouraging professional help.		Training HCPs to engage in myth-dispelling conversations with PWLE and their families by providing educational materials to counter common misconceptions.

### Equality concerns

From a WMM perspective, PWLE shared that equity in treatment was important to them. They voiced concerns about mental health equity, highlighting issues with verbal communication and avoiding labels associated with mental health conditions out of fear of stigma. PWLE also experienced issues with shame or denial, which impeded their efforts to get assistance. To create an inclusive environment, HCPs stressed the significance of treating PWLE equally and placed a strong emphasis on empathy, compassion, and societal awareness. Policymakers emphasised the need to ensure equality between mental and physical health, supporting the integration of mental health services into primary care and the adoption of holistic approaches to healthcare.

*“We experienced discrimination and bullying in the past (before attending this centre)” (PWLE, Female)*.

*“I do not discriminate between patients. The issue of discrimination should be addressed. Discrimination exists. Some service providers believe mental health patients should be treated differently”. (Program Coordinator, Female)*.

*“In the past we used to ask the security officers to watch patients. We then noticed that this upsets patients and that patients felt they were being discriminated against and treated as though they were insane. The way we treat patients has improved a lot. We have made a lot of progress.” (PHC Management, Female)*.

*“There is a lot of discrimination in society. This upsets me.” (PWLE, Female)*.

### Support and compassion

Another aspect of WMM was being treated compassionately by others. Acceptance and support played crucial roles in the mental health journeys of PWLE. Community and family support significantly influenced their willingness to seek treatment. Despite some instances of biased behaviour from specific professionals, most PWLE reported positive experiences due to the empathetic and responsive methods of healthcare providers. Management emphasized the need to support medical staff in recognizing and assisting PWLE, highlighting the importance of empathy in enhancing overall well-being.

*“It is possible that a patient comes in and he is very irritated. He might shout at the staff and say obscene things, we immediately know that he is suffering from MH problems… Usually they are nervous, they might instigate a fight with anyone. You can’t say no to them.” (Data entry officer, Female)*.

### Confidentiality concerns

Maintaining the confidentiality of one’s mental health status emerged as a key priority within the WMM framework. PWLE highlighted how important it is to maintain confidentiality when interacting with healthcare professionals, appreciating quiet discussions and private consultation spaces where they can freely express their ideas and worries without worrying about being judged. Some people specifically asked that their mental health clinical information not be disclosed because they were afraid that their family members and other PWLE at the PHC would judge them negatively. HCPs acknowledged the possible consequences of violating confidentiality and emphasised the significance of preserving a safe and private environment for PWLE to get mental health assistance. Although several healthcare facilities took proactive steps to safeguard the privacy and confidentiality of PWLE, these efforts were occasionally jeopardised by a lack of resources, underscoring the necessity of budgetary allocations to guarantee sufficient privacy protections. Management understood the value of patient privacy in mental health treatment, even in the face of resource limitations, and they pushed for measures to preserve patient information.

*“I keep my condition private. I do not want anyone to know that I am seeing a psychiatrist.” (PWLE, Female)*.

*“Patients tell me that they do not want anyone to know that they are taking psychiatric medication.” (Psychiatrist, Male)*.

### Competency and time management

For HCPs, being competent in their work was an aspect of what matters most to their professional identity. HCPs expressed a need for more time to be devoted to mental health treatment and a desire for more in-depth training and supervision to boost their self-assurance and capacity to give high-quality care. Participation in the mhGAP training significantly increased staff competency. This was in addition to earlier support and guidance from MOPH supervisors. The significance of providing additional time and resources to assist HCPs in providing mental healthcare and resolving staff turnover was stressed by management and policymakers. The importance of supervision in offering continuous assistance and direction was emphasised as being critical to preserving healthcare practitioners’ competency.

*“In the past, supervisors from the MOPH used to offer service providers at PHC centres with a lot of support. Supervisors were providing essential support. We completed the trainings offered by the MOPH a long time ago. Service providers are applying what they know. No trainings are being given at PHC centres at the moment. Service providers might have forgotten the content of the trainings that they received. They have not received trainings in over two years. Supervision is very important”. (Program Coordinator, Male)*

### Willingness

Willingness to help others was identified as a core characteristic of healthcare providers’ professional identity. They showed various degrees of willingness to incorporate mental health services into their jobs at PHC facilities. While some professionals were willing to take on more duties, many were reluctant to do so and would rather refer patients with mental health issues to facilities with specialised training. Issues were brought up, including the requirement for specialised skill sets in order to provide good mental healthcare and interfering with the competence of mental health specialists. Although policymakers emphasised the necessity for designated care coordinators to efficiently manage patient situations and the relevance of PHCs accommodating mental health services, PHC managers demonstrated a limited inclination to modify job descriptions to accommodate mental health integration.

*“In my opinion, I am against GPs overstepping over the psychiatry field.” (GP, Male)*.

*“As a GP, I might interfere with patients suffering from physical problems but not psychological. It is a different specialization that has its experts.” (GP, Male)*.

*“When we asked PHC staff members to provide mental health services, most staff members objected because they did not consider offering mental health support to be part of their job description”. (Program Coordinator, Female)*

### Self-care and burnout perspectives

A key threat to the values what matters most for healthcare workers was burnout, as it hindered their ability to maintain competence and provide compassionate care. Burnout was a subject of differing viewpoints among healthcare providers; while certain individuals expressed satisfaction with their jobs, others recognised its existence and attributed it to elements including excessive workloads and inadequate support. The perspectives of managers about the management of burnout varied, as certain individuals recognised the presence of external pressures but raised doubts about the legitimacy of burnout concerns. On the other hand, others pushed for increasing the level of staffing to mitigate the pressure on team dynamics and patient care.

*“Staff members have not received training on stress management (to help prevent burnout). Knowledge of how to cope with burnout would make a difference.” (Psychologist, Female)*.

*“Staff members do suffer from burnout at times. The patient load is huge because living conditions in Lebanon are very difficult.” (Family Doctor, Male)*.

### Resource sustainability

A lack of resources—both on the part of patients and the healthcare system—posed a threat to the values of WMM, as it undermined healthcare workers’ ability to deliver competent and effective treatment. PWLE highlighted the financial barriers to accessing mental health services, emphasizing the need for affordable options, and addressing additional costs such as transportation and medication. Healthcare professionals have lobbied for the acknowledgment of mental health training days as regular working days to address financial issues. They have emphasised the significance of equitable compensation considering the challenges inherent in their profession. The need of incorporating mental health services into primary care was underscored by policymakers, who highlighted the necessity of training, monitoring, and community mental health services as integral elements. Managers voiced apprehensions regarding the financial limitations that could affect the recruitment of additional mental health experts and the long-term viability of training initiatives.

*“To be able to provide mental health at the level of primary care you need to have a system level approach, so this means that it goes way beyond just training and supervision”. (Policy Maker, Male)*

*“Had we had more time in the centre, we would give the patient more time and we would sit with them for longer…We don’t have time unfortunately.” (Nurse, Female)*.

### Perceptions of stigma

As hypothesized, given that perceptions of patients with mental health conditions threatened the values of WMM to HCPs, we found a number of stigmatizing attitudes related to this group. HCPs exhibited indications of bias and social disapproval, linking specific behaviours exclusively to mental health problems, so perpetuating unfavourable stereotypes. The bias was evident in their prioritisation of effectively managing challenging patients and recognising the influence of mental health on occupational performance and safety. PWLE have emphasised the significant influence of society beliefs on their self-esteem, resulting in feelings of isolation and reluctance to openly communicate their problems due to concerns about being judged and stigmatised. They encountered prejudice, disparaging designations, and ridicule, highlighting widespread societal obstacles. Policymakers acknowledged the significance of enhancing information to tackle stigma but acknowledged that mere awareness may not be adequate. The prevalence of stigmatisation among healthcare professionals was found to be similar to that observed in the general community, indicating that larger societal factors play a role in shaping perceptions of mental health.

*“Some patients do not return after we inform them that they have a mental health condition. They refuse to learn more about their condition.” (Family Doctor, Male)*.

*“Even when we had MH professionals here in the centre, most often the patients would stop mid-way through their treatment.” (GP, Male)*.

### Perceptions of myths in mental health

Myths perpetuated the threats to the values of WMM for both PWLE and healthcare providers. Myths surrounding mental health were prevalent among both PWLE and their community hindering accurate diagnosis and treatment. Concerns were raised by PWLE regarding the prevalence of misunderstandings concerning medications and behaviours, which discouraged individuals from seeking the necessary medical care. For example, one participant reported that their partner discouraged them from taking medication. In the same way, the perspectives of healthcare professionals were notably impacted by cultural and religious views. Certain communities stigmatised mental health conditions as forbidden and favoured prayer over seeking professional assistance. These misconceptions were sustained by these beliefs, which prevented people from seeking psychiatric care, thereby prolonging the diagnosis process, and worsening mental health conditions.

*“My husband told me don’t take medication”. (PWLE, Female)*

*“A mother whose child has psychosis: “Doctor, if he married, would he recover?”” (Psychiatrist, Female)*.

*“Saying that this is their personality, not a mental disorder, is a common misconception.” (Programme Manager, Female)*.

*“Some people assume that the patient is going through difficulties (or has depression) because the patient does not pray. Some religious people think that a person who has faith would not be affected by life’s trials and tribulations. They think that instead of seeing a psychiatrist the patient should pray”. (Nurse, Female).*

### Barriers

#### Organizational barriers

Several organizational barriers hindered efforts to address the perceived threats to the values of WMM for both PWLE and HCPs. The information gathered from policymakers’ interviews highlighted the existence of systemic barriers that posed serious structural challenges and prevented the seamless integration of mental health into primary care. Policymakers highlighted that despite the initiatives to position mental health services as a crucial component of primary healthcare, the current system did not offer a setting that was supportive of this harmonic integration. According to them, this resulted from the novelty of offering mental healthcare at primary centres in the Lebanese context. Mental health was still primarily viewed as a specialized field that should only be handled by professionals, maintaining a separation that hampered holistic healthcare delivery. To address these structural issues, substantial decisions were indispensable. Comprehensive resource allocation was urgently required for crucial elements like community mental health centres, training programmes, supervisory frameworks, and health information systems. The foundation for successfully integrating mental health into primary care was formed by these resources. Such extensive initiatives had the potential to open the door to a more effective healthcare system that cared for everyone’s mental health.

*“A people-centred approach would very much help the integration of mental health support into primary health care.” (Programme Manager, Female)*.

*“I think there are a lot of structures and investments that need to be done in terms of health information systems, trainings, supervisions, and the existence of secondary level mental health centres to support the integration of mental health into primary care.” (Policy maker, Male)*.

## Discussion

### Summary of findings

In our study, we conducted interviews to investigate major domains connected to the WMM framework aimed at reducing mental health stigma in Lebanon. We identified several challenges exacerbating stigma, which align with findings from Gurung et al. [21], who noted the influence of cultural values on stigma in Nepal, which mirrored our observations in Lebanon. Additionally, our findings resonated with the structural barriers identified by Gurung et al. [21], including inadequate mental health policies and insufficient funding. Similar to our findings, a study in Singapore by Tan et al. [22] revealed that stigma impedes help-seeking intentions and affects various aspects of life for individuals with mental illness. The themes underscored the complexity of stigma across different settings, emphasizing the need for culturally tailored anti-stigma campaigns. Based on the findings, we have provided specific recommendations for stigma reduction within the Lebanese primary care system (see Table 2).

**Table 2:** Recommendations for stigma reduction within the Lebanese primary care system

Domain	Recommendations
Healthcare providers	- Engage in continuous training (stigma reduction training, mental health training, etc.) - Participate in initiatives like RESHAPE for social contact with PWLE. - Address and mitigate biases toward individuals with lived experience. - Educate families and trusted adults about mental health
People with lived experience of mental health conditions	- Act as co-facilitators in training for healthcare providers. - Participate in the design, implementation, and evaluation of stigma reduction initiatives.
Management and policy makers	- Invest in mental health policy improvements and funding allocations. - Implement structural changes in healthcare settings to support mental health integration. - Develop community mental health services as part of a comprehensive strategy. - Involve policy adjustments, provide clinical resources, establish redress procedures, and restructure healthcare settings - Support the involvement of individuals with lived experience in stigma reduction initiatives. - Provide training opportunities and evaluate healthcare providers’ competencies through systematic approaches and platforms. (i.e. EQUIP)
Researchers	- Conduct studies to explore the impact of cultural beliefs on mental health stigma - Evaluate the effectiveness of stigma reduction interventions. - Investigate systemic barriers to mental health service integration.

### Discussion of practice strategy

The literature indicated a pervasive stigma against individuals with mental health disorders, not just in Lebanon but also across other Arab nations [21]. Primary care providers have been identified as a source of stigma, with reports that some healthcare providers expressed negative biases toward PWLE [23]. Also, according to the findings of Bhardwaj et al. [24], improving psychological treatment skills and knowledge while incorporating stigma-reduction components into the mhGAP training package may be beneficial for promoting psychological interventions in primary care settings. For instance, the Reducing Stigma among Healthcare Providers (RESHAPE) initiative included social contact with PWLE in the training of non-specialist healthcare workers, which is essential for fostering an inclusive environment [25]. Future investments in stigma reduction within healthcare facilities should prioritize involving stigmatized patients and healthcare providers, addressing both individual and structural levels of stigma [26].

Based on the RESHAPE intervention evaluation, there is likely a relationship between stigma reduction and improved clinical diagnosis accuracy, as well as positive attitude changes [27]. Furthermore, educating families and trusted adults on mental health is crucial to mitigate negative influences on patients’ treatment journeys. Individuals with mental illness are more likely to experience microaggressions from family members, relatives, and healthcare practitioners rather than from strangers or acquaintances [28].

The importance of continuous training and supervision of primary healthcare providers is also vital for effective collaboration with individuals experiencing mental health disorders. The WHO’s EQUIP (Ensuring Quality In Psychosocial and Mental Health Care) platform illustrated the effectiveness of competency-based training in improving care quality for non-specialist providers [29]. The EQUIP platform can effectively assist in the “supervision and support” tasks managed by the MOPH, addressing challenges faced by mhGAP supervisors in overseeing GPs and nurses. It also helps centres manage their trainees, potentially reducing the need for additional supervisory staff from the ministry.

As for the threats to the values of WMM, the misconceptions related to mental health may have perpetuated the notion that mental health conditions were less legitimate or severe than physical illnesses, further marginalizing those in need of support. Myths derived from religion, society, and social customs obscure the truth about mental health. For instance, a study performed in 2015 in Arab countries, clarified the complex relationship of cultural beliefs and misconceptions that contribute to stigma and prejudice against people with mental illnesses [30]. To break stereotypes, fight stigma, and foster an atmosphere of understanding and support, it is essential to address these beliefs through education, awareness, and culturally sensitive approaches. In line with the results of a survey on public attitudes toward mental illness and mental health services in Saudi Arabia. This study demonstrated that traditional beliefs were associated with a negative attitude towards mental health services and individuals with mental illnesses, as well as poor decision-making when seeking help [31]. Therefore, addressing misconceptions was essential for cultivating a more precise and empathetic comprehension of mental health.

### Discussion of structural strategy

In primary care, the integration of mental health services is challenged by systemic barriers. Eaton et al. [32] highlighted the necessity of improving mental health care access in LMICs as part of their research on scaling up mental health services. The results from Bhardwaj et al. [24], highlight the need of ensuring that all primary care facilities have the right structural conditions, such as private rooms for psychological treatment [24]. Even in locations with limited resources, these initiatives could increase the possibility that primary care health personnel will support and facilitate the provision of psychological services [24]. It is to note that the low level of funding allocated to mental health services, have been identified as key challenges for sustainable mental health financing [33].

A complete strategy for resource sustainability in mental healthcare, according to the interviews, should support community mental health services, integrate mental health services into primary care, and provide ongoing training and monitoring. Several important strategies for lessening stigma in healthcare settings were brought to light by the review of several interventions by Nyblade [26]. Prominent measures included “structural” or “policy change” strategies, which involved policy adjustments, providing clinical resources, establishing redress procedures, and restructuring healthcare settings [26].

There are different limitations in this study. Initially, the study was limited to only four PHCs located in Beirut and Mount Lebanon, which may not provide a comprehensive representation of the entire country of Lebanon. Furthermore, the majority of the results are based on self-reports provided by PHC staff and PWLE, which could potentially be influenced by responder bias. The utilisation of the outreaching strategy to communicate with participants via PHCs and focal sites may have had additional effects on the outcomes. Furthermore, the participants’ responses may have been influenced by the fear of losing services and the presence of a predisposition towards societal preferences [16]. Another limitation of the study is the lack of double coding by two independent reviewers; however, the theme extraction was jointly reviewed by the first and last authors to ensure rigor and validity. The study was conducted before Lebanon experienced multiple crises, such as a recession, the COVID-19 pandemic, and the explosion at the Beirut port. These events likely had a significant impact on the healthcare and mental health status in the country.

## Conclusion

In this study, we explored WMM at primary healthcare centre to PWLE, HCPs, policymakers, and PHC management to reducing stigma associated with mental disorders. We identified key domains from interviews to understand WMM and emphasized the implications for reducing stigma. We also highlighted several threats to the values and priorities of PWLE and healthcare provider that further influenced mental health stigma in Lebanon. The WMM framework adapted to Lebanon has a promise for guiding what components are required to reduce stigma in primary healthcare settings.

Future research should evaluate the effectiveness of anti-stigma interventions that actively involve PWLE in both their design and implementation, using tools like the EQUIP platform for ongoing monitoring. Additionally, studies should explore the adaptability and scalability of the WMM framework in different cultural and healthcare settings to enhance its generalizability and impact.

## Electronic supplementary material

Below is the link to the electronic supplementary material.

Supplementary Material 1

## Data Availability

The datasets used and analysed during the current study are available from the first author upon request.

## Abbreviations

WMM: What Matters Most
PHC: Primary Healthcare Centre
HCP: Healthcare Provider
PWLE: People With Lived Experience
NMHP: National Mental Health Programme
MOPH: Ministry Of Public Health
GP: General Practitioner
LMIC: Low- and Middle-Income Countries
mhGAP: mental health Gap Action Programme
EQUIP: Ensuring Quality in Psychosocial and Mental Health Care
