# Supplementary material for: Addressing “what matters most” to reduce mental health stigma in primary healthcare settings: a qualitative study in Lebanon
Source: BMC Prim Care. 2024 Dec 19;25:427. doi: 10.1186/s12875-024-02680-2 (PMC11657562; doi:10.1186/s12875-024-02680-2)
Supplement: Supplementary file 1 — Supplementary Material 1 [file 12875_2024_2680_MOESM1_ESM.docx]

**Consolidated criteria for reporting qualitative studies (COREQ): 32-item checklist**

Developed from:

Tong A, Sainsbury P, Craig J. Consolidated criteria for reporting qualitative research (COREQ): a 32-item checklist for interviews and focus groups. *International Journal for Quality in Health Care*. 2007. Volume 19, Number 6: pp. 349 – 357

| **No.  Item** | **Criteria description** | Study Information |
| --- | --- | --- |
| **Domain 1: Research team and reﬂexivity** |  |  |
| *Personal Characteristics* |  |  |
| 1. Interviewer/ facilitator | Which author/s conducted the interview or focus group? | Lebanon: Research assistants with bachelor’s and Master’s level of education |
| 2. Credentials | What were the researcher’s credentials? E.g. PhD, MD | Lebanon : MD, PhD, MSC, MPH |
| 3. Occupation | What was their occupation at the time of the study? | Lebanon: Research assistant and Service Development coordinators and Public Health Officers |
| 4. Gender | Was the researcher male or female? | Lebanon: Male and female researchers |
| 5. Experience and training | What experience or training did the researcher have? | Lebanon: 3 years research experience |
| *Relationship with participants* |  |  |
| 6. Relationship established | Was a relationship established prior to study commencement? | Lebanon: Previous collaborations between the NMHP team and PHC service providers. No prior relationship with service users and caregivers. |
| 7. Participant knowledge of the interviewer | What did the participants know about the researcher? e.g., personal goals, reasons for doing the research | Lebanon: The participants knew that the researchers were part of the ministry of public health working at the National Mental Health Programme and the reason is to understand Stigma at PHC. |
| 8. Interviewer characteristics | What characteristics were reported about the interviewer/facilitator? e.g., Bias, assumptions, reasons and interests in the research topic | Lebanon: Interest in mental health service research and in line with the objectives of the National Strategy for mental health |
| **Domain 2: study design** |  |  |
| *Theoretical framework* |  |  |
| 9. Methodological orientation and Theory | What methodological orientation was stated to underpin the study? e.g. grounded theory, discourse analysis, ethnography, phenomenology, content analysis | The methods employed included the analysis of previous data sources to identify the WMM framework to reduce stigma against PWLE at primary healthcare centres in Lebanon. In this analysis, we applied the WMM framework to go deeper into the identified themes and determine their significance in guiding culturally grounded anti-stigma interventions. |
| *Participant selection* |  |  |
| 10. Sampling | How were participants selected? e.g. purposive, convenience, consecutive, snowball | Lebanon: Convenience |
| 11. Method of approach | How were participants approached? e.g. face-to-face, telephone, mail, email | Lebanon: face-to-face and telephone |
| 12. Sample size | How many participants were in the study? | N=45 |
| 13. Non-participation | How many people refused to participate or dropped out? Reasons? | Lebanon: Some service users refused to participate because recruitment was done by primary care staff and they reported feeling uncomfortable speaking on topics of stigma with the primary care staff. The number of refusals was not recorded. |
| *Setting* |  |  |
| 14. Setting of data collection | Where was the data collected? e.g., home, clinic, workplace | Lebanon: primary care center |
| 15. Presence of non-participants | Was anyone else present besides the participants and researchers? | Lebanon: no |
| 16. Description of sample | What are the important characteristics of the sample? e.g., demographic data, date | Lebanon: October to December 2018 |
| *Data collection* |  |  |
| 17. Interview guide | Were questions, prompts, guides provided by the authors? Was it pilot tested? | Lebanon: yes, and translated to Arabic |
| 18. Repeat interviews | Were repeat interviews carried out? If yes, how many? | Lebanon: no |
| 19. Audio/visual recording | Did the research use audio or visual recording to collect the data? | Lebanon: audio recording |
| 20. Field notes | Were ﬁeld notes made during and/or after the interview or focus group? | Lebanon: no |
| 21. Duration | What was the duration of the interviews or focus group? | Lebanon: 30 – 45 minutes |
| 22. Data saturation | Was data saturation discussed? | Yes, and additional interviews were conducted to obtain data saturation. |
| 23. Transcripts returned | Were transcripts returned to participants for comment and/or correction? | Lebanon: no |
| **Domain 3: analysis and ﬁndings** |  |  |
| *Data analysis* |  |  |
| 24. Number of data coders | How many data coders coded the data? | One person. |
| 25. Description of the coding tree | Did authors provide a description of the coding tree? | The transcribed data was examined for recurring themes and issues. These were categorized according to their relevance to WMM from the perspectives of healthcare providers, PWLE, centres management and policymakers. Repetitive issues were then grouped under broader domains such as confidentiality, equality, stigma, etc. The thematic analysis aimed to highlight significant areas impacting the delivery of mental health services within the PHC setting. This approach was chosen to give a complete understanding of the underlying causes of mental health stigma in Lebanon, as well as to inform the creation of tailored interventions to reduce stigma in primary healthcare settings. With the WMM framework, the analysis focused on identifying the most significant themes that emerged from the data—those that participants identified as central to their decision-making, values, and behaviours. Moreover, the analysis sought to highlight key concerns and priorities across different key groups, ensuring that the experiences and perspectives of healthcare providers, PWLE, centres management, and policymakers were all considered. In addition, the established framework was applied to identify potential threats to the values of WMM. This involved a detailed assessment of barriers and challenges mentioned during the interviews. The initial analysis, conducted by the first author, focused on identifying and organizing key themes to WMM, threats and barriers to the values and priorities of WMM. This analysis was reviewed by the senior author to ensure accuracy, consistency, and reliability. |
| 26. Derivation of themes | Were themes identiﬁed in advance or derived from the data? | Themes were derived from the data. |
| 27. Software | What software, if applicable, was used to manage the data? | NVIVO was used in the primary data analysis. |
| 28. Participant checking | Did participants provide feedback on the ﬁndings? | Participants did not provide feedback on the findings. |
| *Reporting* |  |  |
| 29. Quotations presented | Were participant quotations presented to illustrate the themes/ﬁndings? Was each quotation identiﬁed? e.g. participant number | Quotations were presented to illustrate major themes and findings. Basic demographic description (gender, participant type) was given for the person making each of these statements. |
| 30. Data and ﬁndings consistent | Was there consistency between the data presented and the ﬁndings? | Yes |
| 31. Clarity of major themes | Were major themes clearly presented in the ﬁndings? | Yes |
| 32. Clarity of minor themes | Is there a description of diverse cases or discussion of minor themes? | Yes |
